# Passive Infrared Motion Sensors Improved the Detection Accuracy of Nocturnal Agitation

**DOI:** 10.1093/geroni/igab046.3422

**Published:** 2021-12-17

**Authors:** Wan-Tai Au-Yeung, Lyndsey Miller, Zachary Beattie, Jeffrey Kaye

**Affiliations:** Oregon Health & Science University, Portland, Oregon, United States

## Abstract

Actigraphy has been used to detect agitation in persons with dementia, although this technology must be worn by participants. Another promising sensing methodology is passive infrared (PIR) motion, which provides continuous, low-cost, and unobtrusive data, and may also improve the detection of agitated periods. Using data from the MODERATE (Monitoring Dementia-Related Agitation Using Technology Evaluation) study, we compared the predictive value of detecting agitation in a male participant, who was 64 years old with Alzheimer’s disease (AD), living in a memory care unit, and monitored with actigraphy on his wrist and four PIR motion sensors within his living quarters. The participant’s medical record indicated that he experienced agitation during 17 nights over 96 consecutive days. 929,037 data points were captured for analysis. From each night, the features extracted from the actigraphy wearable included total and standard deviation of activity counts, activity counts in the most and the least active hours, and median activity counts in one hour. Features extracted from the PIR motion sensors included dwell durations in the areas around bed, sofa, front door and bathroom, and the number of transitions between these areas. Using logistic regression to predict agitated periods, comparable classification performances were achieved using these two sets of features (AUC = 0.74 for wearable and AUC = 0.71 for PIR motion sensors). When these two sets of features were combined, the classification performance showed notable improvement (AUC = 0.83). This study points to the value of utilizing PIR motion sensors for detecting dementia-related agitation.

